# The nature, drivers and equity consequences of informal payments for maternal and child health care in primary health centres in Enugu, Nigeria

**DOI:** 10.1093/heapol/czad048

**Published:** 2023-11-16

**Authors:** Pamela Adaobi Ogbozor, Eleanor Hutchinson, Catherine Goodman, Martin McKee, Obinna Onwujekwe, Dina Balabanova

**Affiliations:** Department of Psychology, Enugu State University of Science and Technology, PMB 01600, Agbani, Enugu, Nigeria; Health Policy Research Group, College of Medicine, University of Nigeria, Enugu Campus, PMB 01129, Enugu, Nigeria; Department of Health Services Research and Policy, London School of Hygiene & Tropical Medicine, 15-17 Tavistock Place, London WC1H 9SH, United Kingdom; Department of Health Services Research and Policy, London School of Hygiene & Tropical Medicine, 15-17 Tavistock Place, London WC1H 9SH, United Kingdom; Department of Health Services Research and Policy, London School of Hygiene & Tropical Medicine, 15-17 Tavistock Place, London WC1H 9SH, United Kingdom; Health Policy Research Group, College of Medicine, University of Nigeria, Enugu Campus, PMB 01129, Enugu, Nigeria; Department of Health Administration and Management, University of Nigeria, Enugu Campus, PMB 410001, Enugu, Nigeria; Department of Health Services Research and Policy, London School of Hygiene & Tropical Medicine, 15-17 Tavistock Place, London WC1H 9SH, United Kingdom

**Keywords:** Informal payments, maternal and child health, equity, primary health care, user fees

## Abstract

In Nigeria, most basic maternal and child health services in public primary health-care facilities should be either free of charge or subsidized. In practice, additional informal payments made in cash or in kind are common. We examined the nature, drivers and equity consequences of informal payments in primary health centres (PHC) in Enugu State. We used three interlinked qualitative methods: participant observation in six PHC facilities and two local government area (LGA) headquarters; in-depth interviews with frontline health workers (*n* = 19), managers (*n* = 4) and policy makers (*n* = 10); and focus group discussions (*n* = 2) with female service users. Data were analysed thematically using NVivo 12. Across all groups, informal payments were described as routine for immunization, deliveries, family planning consultations and birth certificate registration. Health workers, managers and policy makers identified limited supervision, insufficient financing of facilities, and lack of receipts for formal payments as enabling this practice. Informal payments were seen by managers and health workers as a mechanism to generate discretionary revenue to cover operational costs of the facility but, in practice, were frequently taken as extra income by health workers. Health workers rationalized informal payments as being of small value, and not a burden to users. However, informal payments were reported to be inequitable and exclusionary. Although they tended to be lower in rural PHCs than in wealthier urban facilities, participant observation revealed how, within a PHC, the lowest earners paid the same as others and were often left unattended if they failed to pay. Some female patients reported that extra payments excluded them from services, driving them to seek help from retail outlets or unlicensed health providers. As a result, informal payments reduced equity of access to essential services. Targeted policies are needed to improve financial risk protection for the poorest groups and address drivers of informal payments and unfairness in the health system.

Key messages

- Informal payments for maternal child health services are very common and highly regressive in primary health centres, particularly affecting the ability of poorer patients to access health care.
- Informal payments are collected with the objective of generating funding for supplies and covering shortages, to enable the health facilities’ operations, but in practice, these are often used to augment the personal income of health workers.
- The institutional drivers of informal payments are complex, including absence of clear and transparent regulations about official payments, lack of oversight, pressure from health facility managers, lack of automated systems for formal payments, and most importantly, lack of funding.
- There is a need to address informal payments in order to improve equity of access, but this requires understanding their patterns and addressing multiple institutional and health worker-related drivers.

## Introduction

Equitable access to maternal and child health care (MCH) services is essential if countries are to reduce the maternal mortality rate (MMR), a global priority, and while there has been progress there is still much to do, especially in low- and middle-income countries (LMICs) (Alkema *et al.*, 2016; Bongaarts, 2016; Mehboob *et al.*, 2021). The Sustainable Development Goals call for a reduction of MMR to below 70 per 100 000 live births by 2030 (WHO, 2022) but the most recent data from Nigeria, from 2019, report a figure of 917 (WHO, UNICEF, UNFPA, World Bank Group, and the UN Population Division, 2019), which is among the worst in Africa.

Primary health centres (PHCs) in Nigeria offer access to skilled birth attendants, family planning services, antenatal care and community management of acute malnutrition and communicable diseases. These are described in detail in documentation produced by the Free Maternal and Child Health Programme, which provides free antenatal care, delivery, medical care up to 42 days after delivery, family planning, routine laboratory investigations and treatment for children under 5 years of age in state facilities (Ogbuabor and Onwujekwe, 2018; Eze *et al.*, 2020). However, in 2015, the list of services provided free or charge was scaled back due to shortage of funds (Onwujekwe *et al.*, 2019). The transition from free to partially free care was incremental and was not announced publicly, with no documented official regulatory framework in place stating what should be paid and at what level. The officers in charge (OIC) of managing facilities were left to cope with the disruption of funding without formal guidance on the amounts to be paid for delivery, based only on informal communications when health workers requested funding. No official document listing what fees should be paid, and what exemptions should be made, was provided to facility managers. At the same time, announcements continued to be made through the media encouraging people to access free MCH services in PHCs. Throughout this period there was considerable concern that informal payments were commonly being charged for both MCH services that should have been free and those for which modest charges were (informally) allowed. The situation subsequently changed with the introduction of the Basic Health Care Provision Fund (BHCPF) in 2022 in Enugu State but this was not rolled out in the facilities under study until after our data collection was completed in 2021. The BHCPF was established under Part 1 Section 11 of the *National Health Act* as a means to fund universal access to primary health care but, especially, for the poor (National Primary Health Care Development Agency, 2023).

Informal payments are payments in cash or in kind over and above official user fees. The evidence on whether informal payments are regressive is mixed. Some authors have suggested that informal payments could lead to quasi-redistribution among patients (Kessel, 1958; Szende and Culyer, 2006). In these cases, locally knowledgeable, and sympathetic, staff are presented as playing a ‘Robin Hood’ role by subsidizing the poor at the expense of the rich (Kankeu and Ventelou, 2016). However, other studies have shown that informal payments are demanded by staff regardless of a patient’s ability to pay (Nekoeimoghadam *et al.*, 2013; Srivastava *et al.*, 2015). In MCH services, informal payments appear regressive, posing an important barrier to service utilization (Skordis-Worrall *et al.*, 2011; Srivastava *et al.*, 2015), and widening inequalities in access to health care (Stepurko *et al.*, 2010).

Recent debate on reducing the burden of informal payments suggests that interventions need to be based on a deep understanding of how informal payments are manifested in different contexts (Binyaruka *et al.*, 2022; Ramesh *et al.*, 2022). This paper uses qualitative research data to explore how and why informal payments are demanded and made for MCH at PHCs in Nigeria, with a particular focus on their impact on the poorest patients seeking care.

## Methods

### Study setting

The study was carried out in Enugu State, in southeast Nigeria. The state has a population of 3.3 million with an annual growth rate of 2.8% (Federal Republic of Nigeria, 2010) and a mix of urban and rural areas. In 2022, 85% of the population lived below the poverty line (CHORUS Nigeria, 2022), a large increase over recent years from 58.13% in 2019 (Nigeria Bureau of Statistics, 2019). MCH care is provided by PHCs, which are staffed by nurses, midwives, community health officers, health technicians and community health extension workers, while doctors may cover three or more PHCs. The PHCs are overseen by a local government authority but also receive support from State institutions. They are managed on a day-to-day basis by a PHC coordinator, who supervises the facility officer in-charge (OIC) and who is subordinate to the politically appointed supervisory councillor responsible for health, who in turn reports to the local government area chairman (Adeyemo, 2005; Abdulraheem, 2012). There are also ward development committees (members of the community) that foster community engagement with PHCs (Federal Republic of Nigeria, 2014) and whose chairman must be a member of the health facility committee (HFC). HFCs are committees that oversee and support PHCs, comprising health workers, community leaders and respectable community members. However, PHCs are often characterized by weak governance and limited accountability (Ogbozor *et al.*, 2022). They also suffer from a lack of clinical staff with appropriate skills, especially in rural areas where need is greater and funding unpredictable.

### Study design and data collection

We employed a sequential approach using a mix of qualitative research methods (Brewer and Hunter, 2006; Bazeley, 2012; Anguera *et al.*, 2018). The ethnographic participant observation took place over eight weeks, from January to March 2021. This was followed by in-depth qualitative interviews (IDIs) and finally focus group discussions (FGDs) to elicit the nature of informal payments, degree of equity and perceived burden. We purposively selected 4 of 17 LGAs (2 urban and 2 rural)—Enugu North, Enugu East, Nkanu East and Nsukka. Six PHCs and two LGA health departments were selected intentionally across the four LGAs to cover facilities experiencing high and low volumes of clients as we anticipated that the intensity of patient activity might be associated with differences in the frequency of corrupt practices.

### Participant observation

Participant observation in PHCs and LGA health departments was conducted by four experienced social science researchers familiar with the context. Each focused on two sites—two PHCs or one PHC and one LGA health department. The first two weeks of observations were unstructured, prioritizing identification of corrupt practices and accountability issues that pose a particular burden on service users. The following six weeks were more structured, based on decisions about where and whom to focus on during weekly supervisory meetings with a senior anthropologist, economist and health systems researcher. Observation included: (1) following patient journeys through the health facility (Mogensen, 2005); (2) following health workers through their everyday practice over the course of a day or week; and (3) observing social relations and overall context of activities at study sites. Brief field notes and sketches were made during the observations, which were written up at the end of each day. Alongside observations, the researchers engaged in unstructured discussions with health workers/managers and service users to clarify practices and to gain a deeper understanding of behaviours. This exploratory work led to identifying informal payments as a particularly common problem affecting service users. Supervisory meetings were also used to reflect upon the position of the researchers in the field, and in particular how staff and patients/care seekers were responding to our presence. We noted how patients often thought research staff were health workers and we ensured that we always took time to explain our position as researchers to the patients. At the beginning of the research, some health workers could be suspicious about why we were at the facilities. We took time to talk to all health workers about our role as researchers. As the fieldwork unfolded and especially after week 4–5, staff appeared at ease with our presence and with discussing their practice (including around informal payments) during both formal and informal interviews.

### Qualitative interviews

Interviews were used to gain a deeper understanding of informal payments alongside other corrupt practices that emerged during our observations. Data were collected using two pre-tested IDI and FGD guides. The first IDI guide was used for health workers and their managers, and the second for policy- and decision makers. The FGD guide was used for female service users. The guides elicited information on governance and accountability processes: rules and operational guidelines on informal payments, charges for MCH services, and experiences accessing MCH services. The topic guides drew on previous research on drivers of corrupt practices in Nigeria’s health system (Onwujekwe *et al.*, 2020), expert advice, and our observations of informal payment practices made during earlier fieldwork. As the ethnography progressed, more questions were added to explore additional activities observed, understand their rationale and triangulate findings with policy makers. Participants were interviewed at different locations based on their preference to enable them to freely express themselves.

The IDI respondents were purposely selected based on their roles and involvement in providing and coordinating health services. Participants comprised 19 frontline health workers (5 midwives, 3 nurses and 11 community health extension workers), 4 PHC managers (officers in charge), 10 policy makers (4 heads of health department, 1 supervisor for health, 2 LGA immunization officers, 1 LGA chairman, and 2 senior officials from the State Ministry of Health and Enugu State Primary Health Care Development Agency). Written consent was obtained from those participating in the IDIs before the interview commenced. The two focus group discussions were held with 20 purposively selected female service users who had accessed MCH services in the previous year. There were 10 women in each FGD. One of the researchers recruited participants by visiting homes within the PHC community, with the help of a local guide. The purpose of the research was explained briefly and those expressing an interest were provided with a more detailed explanation of the objectives of the study, any benefits and risks, the voluntary nature of participation, and the confidentiality of data provided. Those who consented, in writing, were invited to a discussion. Table 1 presents the socio-demographic profile of IDI and FGD participants.

**Table 1.** Socio-demographic profile participants in-depth qualitative interview respondents

Category	Role/designation	Male	Female	Below 40 years	Above 40 years
Policy makers	Senior manager, SMOH	1	–	–	1
	Senior manager, SHPCDA	1	–	–	1
	LGA chairman	1	–	–	1
	HOD	1	3	–	4
	Supervisor for health	1	–	–	1
	LGA immunization officer	0	2	–	2
PHC health managers	Officer in charge	0	4	–	4
Frontline health workers	Midwife	0	5	4	1
	Nurse	1	2	1	2
	S.CHEW	0	4	1	3
	CHEW	0	7	7	0
FGD Participants					
	Women who assessed MCH services in the last 1 year	0	20	20	–

SMOH, State Ministry of Health; SPHCDA, State Primary Health Care Development Agency; HOD, Head of department, LGA, Local government area, CHEW, Community health extension worker; S.CHEW, Senior community health extension worker; OIC, Officer in charge.

With participant observation, researchers obtained informed consent from heads of health departments and facilities. All interviews were conducted in English except the FGDs, which were in Igbo, the language indigenous to the region. All interviews were audio-recorded and fully transcribed. The FGDs conducted in Igbo were transcribed verbatim and translated into English by bilingual members of the study team.

### Data analysis

Findings from the participant observation were discussed each week during the supervisory meeting to identify emerging practices and behaviours. Field notes were read to ensure clarity and analysed inductively by the four field researchers, leading to the generation of inductive codes and themes, using NVivo 12. A narrative synthesis of the findings was undertaken around three broad themes: (1) patterns of informal payments across MCH services; (2) exemptions from informal payments for the vulnerable and poor; and (3) institutional drivers of informal payments.

### Ethical approval

We drew on the Association of Social Anthropologists ethical guidelines (Association of Social Anthropologists (ASA), 2019). In addition, we were influenced by Nancy Scheper-Hughes’ idea of companheira and the rejection of non-involved ethnographic practice as an ethical position (Scheper-Hughes, 1995). Following Scheper-Hughes, in this text we have identified where we ‘participated’ more fully in the life of the PHCs (e.g. by paying for a young woman in order that she could leave the facility having given birth). We developed an extensive standard operating procedure with a traffic light system that indicated how the researchers should react to any situation of coercion of patients, threats, etc. with specific procedures to be followed and emergency contacts to report incidents. Ethical approval was obtained from the authors’ institutes.

## Results

### Patterns of informal payments across MCH services

In all of the PHC facilities included, we consistently observed health workers soliciting informal payments for essential MCH services including routine immunization, family planning, delivery and birth certificate registrations. In addition, most women coming for delivery were required to bring with them additional supplies of disinfectant, toilet paper, gloves and plastic sheets.

The size of informal payments varied across PHCs. We observed that informal payments tended to be higher in PHCs located in urban areas with higher MCH utilization. Many health workers said that they saw residents in urban areas as more likely to be in high and middle economic classes and so, able to pay higher informal payments. By contrast in rural areas, health workers felt that women could only pay lower charges.

We present further details on the pattern and justifications for informal payments by service.

#### Routine immunization

We witnessed health workers charging payments informally at all the facilities we worked in, ranging from (Naira) ₦ 100 to 150 (US$0.38) per visit, and this was routine. In one urban facility, the deputy OIC immunized babies with the support of two interns. As women carrying their babies approached, they checked the immunization card to confirm the child’s age. The intern would hand the vaccine over, the deputy OIC would administer it to the baby and advise the mother to give the baby paracetamol at night if necessary. Each mother then dropped ₦ 150 (US$0.40) into a cardboard box next to the deputy OIC with no discussion about the cost or whether they would have to pay (Research notes 2021, participant observation, urban PHC).

Health workers argued that informal payments for vaccination were necessary to cover the cost of obtaining vaccines at the LGA health department. Although they should have been supplied free of charge to the PHCs, in practice there was a bi-yearly charge made at vaccine collection points for reasons that were unexplained. Health workers felt that it was reasonable to collect a modest fee and it was not a burden for most users.

*We are collecting* ₦ *100 (US$ 0.25), which is not so big. If we say let’s charge for immunization, it will be higher because they charge us money at the LGA cold room, where we carry the vaccines. We pay between* ₦ *200 and 2000 (US$ 0.5–5) depending on the quantity of vaccine. We pay every 6 months, once you get there, they will check if you have cleared your old debt. So, we are collecting* ₦ *100 to cover the money we pay.* (IDI, health worker)

In informal discussions and interviews, health workers and their managers argued that informal payments for immunization also generated revenue needed for relevant consumables required for the procedure (cotton wool, gloves and/or syringes), which were not consistently supplied free to PHCs. Finally, health workers described how the revenue generated was needed to pay ‘volunteer’ medical and non-medical staff employed on an ad hoc basis by the PHC rather than on stable contracts. All health workers and managers described the importance of employing volunteers because of ongoing staff shortages as a consequence of the failure of the State government to recruit PHC health workers over the past 5 years. The use of volunteers and other ‘ad hoc’ workers (e.g. staff working elsewhere but covering gaps) was encouraged by LGA health managers but no additional money was provided for payment of volunteers. They are employed by PHC managers unlike the ad hoc staff who are employed and deployed by the LGA. Engagement of volunteers is widespread across PHCs in Enugu State. PHC managers are responsible for paying volunteers stipends.

*People keep saying that we charge for immunization, but we need to make extra cash because we have to pay the volunteers [i.e. ad hoc staff] supporting us, there is shortage of staff due to high patient flow. The LGA does not pay 14 volunteers that support us in both clinical and non-clinical duties, we pay them.* (IDI, OIC)

#### Family planning

Family planning commodities and consumables are supplied free or charge to PHCs by the Federal Ministry of Health and, sometimes, by non-governmental implementing partners. The service is expected to be free for users but in practice we observed health workers charging all patients for family planning products and consumables used for their administration. As with immunization, informal payments appeared routine in all the facilities where we conducted participant observation.

Informal payments for family planning varied more than those for immunization, ranging from ₦ 200 to 3000 (US$0.5–7.5) depending on the type of commodity provided, with higher amounts charged for contraceptive implants but less for injectable and oral pills. For example, in one urban family planning unit, a researcher witnessed women receiving injectable contraceptives for ₦ 250 (US$0.63), oral pills for ₦ 500 (US$1.25), and an intrauterine device for ₦ 1500 (US$3.75). In discussions, the OIC reported that these were standard charges and that they also offered an implant for ₦ 2000 (US$5), and charged an extra ₦ 1000 (US$5) to remove a broken implant.

Health managers argued that these charges were necessary to enable them to procure the consumables—iodine, gloves, cotton wool, etc. that are routinely used when providing family planning services. A health manager explained:

*Family planning is not free. To practice and maintain a free treatment is very difficult because what they (government) made to be free is the commodity and not the service, and the commodity is nothing without one rendering the service. You can give drugs for free, but if it is an implant or intrauterine device, you have to collect a token for syringe, plaster, iodine, cotton wool, and Xylocaine.* (IDI, OIC)

Another reason given for charging for family planning was the irregular supply of family planning commodities, which meant that PHCs had to procure them from retail outlets or wholesale drug shops to avoid stock outs.

*Presently, we don’t have injectable, I bought it because if clients come, you keep on prescribing drugs to be bought elsewhere, and they won’t come again. I buy these from my little income. I will add little money while selling it to patients that will be affordable to everybody*. (IDI, OIC)

#### Child delivery

Whereas informal payments for family planning and immunization were made in cash, informal payments for facility deliveries took the form of requests that pregnant women bring commodities. These included gloves, disinfectant (e.g. Jik, Dettol), tissue paper, sanitary pads and plastic sheets; and were in excess of those that an individual would need for their own care. During the IDIs, health workers reported that the use of surplus commodities was determined by the OIC—typically a few were kept for use at the facility by health workers, some were sold to pregnant women who failed to bring all the items required for delivery, while others were shared by health workers on a monthly basis for their personal use. Most OICs complained that sometimes they were asked to send the proceeds (money and toiletries) to health managers at the LGAs on an informal basis. Formal delivery costs varied, ranging from ₦ 7000 to 20 000 (US$17.5–50.0) at urban PHCs and from ₦ 7000 to 10 000 (US$15–23) at rural PHCs. Women who could not pay were attended to when in labour but then detained at the facility until all payments were made. We observed this during participant observations at several study sites and on one occasion the research team contributed payment for a woman abandoned in the hospital by her husband for a week.

A female FGD participant explained that the additional costs of giving birth encouraged women to miss appointments and stay away from the health facility.

*I was charged 10 000 Naira ($25) for delivery last year I gave birth in the PHC and I feel it’s too much. I came with all items listed in my list. Some women cannot afford this and will choose to give birth at home with the help of local woman who deliver babies (traditional birth attendant).* (FGD participant, urban)

However, a nursing mother participating in an FGD said that the delivery items and costs were not too high when compared to what is charged in private hospitals:

*I don’t feel it’s too much. There is a place you would go for delivery and you see the place is dirty and smelly, you wouldn’t like it. When you enter labour room in PHCs, you perceive drops of Dettol or Jik (disinfectant) than smell of blood. The items are not much, though they don’t use all immediately for the patient.* (FGD participant, urban)

#### Birth certificates

In Enugu, birth certificates are issued in busy PHCs either daily or during immunization days by staff from the National Population Council. Certificates are meant to be issued free of charge, but we observed fees being charged frequently, with apparently no fixed price at any facility. In one facility, the man issuing certificates did not charge for them but only requested a tip at the end of the service. In others, we witnessed parents paying ₦ 100 to 2500 (US$0.13–6.25) for newborn babies’ certificates, and for older children (8 years and above) ₦ 2000—₦ 10 000 (US$5–25) or more. These costs were lower in rural PHCs where charges for babies were ₦ 100 (US$0.25) and ₦ 2000 (US$5), respectively. The charges appeared to be at the discretion of the person issuing the certificates and based on their perception of the user’s wealth, but they were never free even for the poorest users. In two different urban PHCs, we witnessed a range of reactions from people seeking certificates. Some agreed to pay, some provided payment and a tip, but some with insufficient funds left without obtaining their certificates.

This prompted our researchers to confirm from health workers and managers that birth certificates should be free (according to the regulations), but also that some contract staff posted to PHCs usually would charge money, at their discretion, to complement their wages and cover transport costs.

*Birth certificate should be given free and government announces it to be free but some staff use to collect money from people like ₦ 200, ₦ 300 or ₦ 5000 (US$ 0.5–12.5) depending on individual. This man here only asks people to tip him but not imposing it on them unlike the other chubby woman, who collects minimal of ₦ 500 (US$ 1.25) or nothing. Birth registration from birth to 18 years is free now*. (IDI, community health extension worker)

### Exemptions from payment of informal payments for the poor and vulnerable

Participant observations and FGDs confirmed that informal payments for MCH services were applied universally, with very few or no exemptions though in some cases, users could bargain. The rich appeared to be paying the same amounts as the poor, and we never witnessed poorer patients being exempted from payments during the eight weeks of observation. In addition, the majority of FGD participants reported that some poor people were denied medical services if they were unable to pay, sometimes forcing them to seek alternative treatment from drug shops or traditional birth attendants without a doctor’s prescription, to self-medicate, or to forego care, potentially with fatal consequences.

This is illustrated by the following case. One afternoon a mother brought a malnourished child to a rural facility. She paid for the immunization but was criticized by the OIC when she said she was unable to purchase paracetamol to give to the child if she developed a fever later in the day. As she left the facility, the mother described her discomfort at attending the facility when things were ‘rough for her family’ (meaning when they had no money). She thought that the nurses liked to prescribe expensive medicines for which patients have to pay a considerable amount of money and she preferred to go to patent medicine vendors to obtain cheaper medicines (Research notes 2021, participant observation, rural PHC).

Female service users participating in the FGD also described their perception that the prices for delivery and MCH services were high. This meant that when they could not afford to pay, they did not attend PHCs but stayed at home and self-treated. They reported that health workers at PHCs provided them with medication commensurate with the money they had and/or were willing to pay and that in an emergency, the patient would be treated but detained until their relatives or an NGO paid.

### Institutional drivers of informal payments

In-depth interviews with state-level health policy makers, managers and health workers revealed the major challenges confronting PHCs as they tried to meet the health needs of the population with limited resources. These challenges drove health workers to solicit informal payments from patients, while policy makers failed to take action to prevent them. We now describe these challenges further.

#### Underfunding of primary health-care facilities

PHCs in Nigeria have been neglected for many years. Their funding should flow from the States through the LGAs to the PHCs, but as the LGAs are autonomous, these funds are often disbursed according to the interests of the LGA chairman, with no effective accountability.

*When you organize primary health-care activities at the local government level, you will have no coordination, no articulation, and in fact, no result. This is because new local government chairmen usually prefer to carry out the same project in their village, thereby abandoning the one already started by another chairman elsewhere and this is related to politicization of health and we are trying to fight.* (IDI, policy maker)

This view was widespread among health workers and managers who were interviewed, although they also pointed to a failure of communities to support their local PHCs.

*In every LGA budget, funds are allocated to health sector but most times they are not disbursed. LGA chairmen support health if he has interest, if not, nothing will be done in health sector.* (IDI, head of department)

#### Pressure on facility heads from health managers

Despite acute underfunding of PHCs, some health managers complained of being forced to remit a certain percentage of user fees directly to the State Ministry of Health every month. The OICs also described being forced to send proceeds from sales of items such as Dettol, other sterilizing liquids, and tissues. Non-compliance with these informal demands could lead to OICs being transferred to remote rural areas or other sanctions. As one OIC stated:

*The HOD will call you that she needs money for something, f you don’t give, she will not be happy. She will tell you that the PHC is always busy, you should find a way to be making money. Sometimes, she might not say it but it’s shown in her attitude by denying you any benefits from LGA and she might transfer you on the ground that you are not servicing your table well, she will being a capable person. This is very unofficial and informal.* (IDI, OIC)

#### Inadequate oversight

Most health workers and managers complained of irregular external supervision by State and LGA teams, which allows OICs and health workers to engage in a range of corrupt practices (absenteeism, charging extra delivery supplies, and costs for free services).

*Supervision is key, they will correct a lot of anomalies if they can go across the facilities. It could be that funds are no more coming for supervision because all they are after is data and not quality life.* (Health worker, urban PHC)

The lack of meaningful community oversight of the affairs of the PHCs was also viewed as contributing to more frequent informal payments. There was little or no involvement of HFCs. We observed, and health workers who we interviewed confirmed, that the absence of such community involvement allowed the OIC to enforce payment of informal payments.

#### Lack of an automated payment system

Payments for services were made in cash and there was no electronic system or issue of receipts that would facilitate accountability. During participant observation, we noted that no information was displayed for patients about what the official payments should be. This created an information asymmetry as patients were unaware of which services were free. Most FGD participants reported that they did not receive receipts or a breakdown of payment or any explanation after paying for PHC services.

*I have been using this PHC for over 3 years now. There is nothing like giving receipts after paying. They will only tell you the total charge and not the breakdown. I will pay and go with my drugs.* (FGD participant)

## Discussion

To our knowledge, this is the first study exploring patterns of informal payments for MCH services in PHCs in Nigeria. We have found that informal payments are extremely common, both for MCH services that should be provided free of charge, and for those where there is an official charge. Although these practices are illegal, they appear to be accepted as routine by health workers and managers and either tolerated or expected to some degree by service users. Informal payments were found to be quite variable across facilities and appeared to be set at the discretion of health workers or, for birth certificates, at the discretion of visiting National Population Council staff.

A strength of this study was the combination of ethnographic participant observation and qualitative interviews (FGDs and IDIs). One of the key challenges in studying informal payments is that it is an illegitimate activity, so it may be hard to gather data on practices through interviews/surveys due to social desirability bias. Our extended periods of ethnography allowed us to develop a rapport with facility staff, and for them to become accustomed to our presence, increasing the likelihood that we could observe their normal practice. However, we cannot rule out the possibility that even more extensive informal payments would have been charged if we had not been present. A further strength is the inclusion of a range of different respondent categories, from service users to health workers, health facility managers, State and LGA policy makers, allowing us to get a holistic view of the drivers and implications of informal payments. In terms of limitations, we only worked within six PHCs and four LGAs, and these were all located in one state/geopolitical zone. Care must therefore be taken in generalizing these findings to MCH care in other areas of Nigeria.

A similar study that examined informal payments for treatment of malaria in six LGAs in Anambra State, Nigeria, also revealed high levels of informal payments, which increased the economic burden associated with the disease (Onwujekwe *et al.*, 2010). However, given the importance of the topic, similar studies are needed in other settings and for other types of health service.

Our findings of widespread informal payments for routine immunization, birth certificate registration, deliveries and family planning services are in line with some other studies on MCH in LMICs. For example, women in Tanzania and Kenya were charged for family planning injectable contraceptives and implants, which were meant to be free in government hospitals (Radovich *et al.*, 2019; Busse *et al.*, 2022). This is consistent with our study, and although we found patients were charged for all family planning commodities, prices varied. Afsana (2004) found that informal payments were common when seeking obstetric care in hospitals in Bangladesh, making women afraid to seek help lest they incurred huge costs if they required additional services, such as a caesarian section, laboratory investigations, blood transfusions or assistance by doctors in delivery. Informal payments, especially for MCH services and malaria, have been reported to cause a significant financial burden and catastrophic health expenditures for familiesin developed and developing countries including Nigeria, forcing them to spend their savings or borrow (Afsana, 2004; Sharma *et al.*, 2005; Onwujekwe *et al.*, 2010; Balabanova *et al.*, 2012). We did not find any ‘exemptions’ for poorer people, even though health managers said that procedures did exist for formal payments but these were complex and the rules unclear, limiting their application, although health facility managers did have some discretion. In effect, everybody paid regardless of their personal circumstances. Similarly in Tanzania, the poorest were as likely to be charged informally for family planning as the richest (Busse *et al.*, 2022).

The study extended our knowledge by challenging the ‘Robin Hood’ hypothesis, although this was not an objective of our study, as it would have required a larger quantitative data set. Originally suggested by Ensor and Savelyeva (1998) as applying to informal payments, this has subsequently been examined in Africa by Kankeu and Ventelou (2016). In brief, it proposes that physicians seek different levels of informal payments based on the perceived ability of the patient to pay, thereby providing a degree of redistribution from rich to poor. What we did observe, and noting the caveat that we could not quantify payments, was that while informal payments seemed both more common and higher in the urban areas where wealthier people live and lower in poorer rural areas, there was no mechanism for cross-subsidization among facilities. Nor did we see evidence that poorer people paid less. Instead, they were denied care, driving them to retail outlets or unlicensed health providers.

The situation we observed is problematic on several levels. First, the scale of informal payments and their regressive nature will make it even more difficult to reduce MMR and achieve the corresponding Sustainable Development Goals to reduce the global maternal mortality ratio to less than 70 per 100 000 live births (WHO, 2022). Second, the financial burden will increase the risk of impoverishment, with consequences for the family and community. Third, charging for birth certificates undermines the child’s right to identity and thus their ability to access necessary services and opportunities in the future.

We saw that the problem has many more drivers than simply health workers seeking to enrich themselves. There are institutional and systemic issues that allow informal payments to thrive. These included the absence of clear and transparent regulation of which services should incur fees and how much should be paid, a lack of oversight, pressure from health facility managers, a lack of automated systems for formal payments, and perhaps most important, a lack of funding for PHCs. A sharp decline in the funding of PHCs after 2015 means that in recent years, PHCs were allocated no funds for their operational activities, and were expected to generate their own revenue. In order to survive, PHCs had to levy charges. This was done with little formal guidance or transparent policy, although there are indications from the in-depth interviews with PHC managers that this was regulated by informal agreements between PHC managers and their superiors at state level. The lack of clear rules, may have created greater leeway to charge informal fees, generating perverse incentives to provide inappropriate care. Informal payments were seen by health workers and managers as a mechanism to generate discretionary revenue to cover their operational costs. Thus, there were incentives to avoid setting a clear and uniform policy, instead giving PHC facilities some autonomy in obtaining additional revenue to fill gaps. In a review of drivers of informal payments in maternal care, Schaaf and Topp (2019) also found that informal payments frequently comprised a significant portion of the operational funding for health facilities, and were generally collected and spent at the discretion of facility management, mainly for goods and services that supported patient care such as petrol, payment of ad hoc staff, and procurement of consumables for administering some MCH services like family planning and immunization (Olivier de Sardan, 2011; Diarra, 2012; Schaaf and Topp, 2019). Olivier de Sardan (2011) described this as informal privatization; health workers can use informal payments to make the system work but it becomes a racket benefitting the providers to the detriment of the service users and especially the poor (Gaal *et al.*, 2006; Olivier de Sardan, 2011; Diarra, 2012).

Another key driver of informal payments was inadequate oversight from both superiors, reflecting infrequent supportive supervision, and from community members, who had very limited involvement in monitoring the activities of health workers. Other studies in LMICs have shown how weak supervision and oversight create opportunities for rent seeking (Fjeldstad, 2004). In Tanzania, a pro-poor exemption policy, accompanied by high levels of supervision and oversight by health managers and local government health staff, reduced informal payments (Maluka, 2013; Binyaruka *et al.*, 2021). By contrast, at our study sites, LGA managers were well aware of the widespread charging and even demanded that OICs pay them a portion of their internally generated revenue, actively encouraging higher fees. In this context it is very unlikely that greater monitoring by immediate supervisors and communities alone will be able to hold health workers accountable or enforce any penalties on offenders. Moreover, the HFCs who are meant to improve service accountability have limited power to act.

Addressing informal payments is complex as the problem is systemic and the context needs to be carefully considered. Our findings point to a need for collaboration between authorities at all levels, from the State through the LGA to the facility and the communities. This should include a mix of incentives and sanctions appropriate to the context (Ogbozor *et al.*, 2022).

The health reforms currently being implemented in Nigeria, including the introduction of the BHCPF in Enugu State, do not include a specific focus on reducing informal payments, which may thus undermine the anticipated expansion in MCH coverage. Addressing informal payments in MCH is likely to require a multi-dimensional approach, with a strong focus on the poorest users. Fundamentally PHCs need to be provided with a legitimate source of funding for covering their operational costs to ensure that it is possible to provide services without informal payments. Financing PHCs has been a core challenge for the Nigerian Government, with health budgets at the federal level decreasing since 2012 (Uzochukwu *et al.*, 2018; Federal Ministry of Health, 2015, 2023). The financial allocations at LGA level do not extend beyond the payment of salaries, and budgets are not earmarked, leading to delays in the release (or at times non-release) of PHC funds (Uzochukwu *et al.*, 2018). Strengthening accountability and proper accounting for the use of PHC funds is a high priority for both governments and donors.

To reduce political interference at state and local government level, we propose leveraging on the accountability framework for implementation of the BHCPF (National Primary Health Care Development Agency, 2021), advocating for direct flow of funds from federal to state to facilities where an operational budget is transferred directly into a PHC bank account with the facility manager and a community representative, usually the chairman of the ward development committee acting as signatories for the account. We propose an expansion of the BHCPF funding mechanism to all PHCs in Enugu and Nigeria, operated in the model of the State’s Health Insurance Scheme, with the goal of giving rural residents affordable, comprehensive and efficient health care including free MCH services and eliminating informal payments.

To bring about a change in health worker behaviour, these strategies would need to be accompanied by public awareness-raising of their rights to a range of free MCH services and the appropriate charges for other services, through both public display of charges at facility level, and broader communications campaigns. These strategies need to be supported by supervision and effective accountability structures at all levels of the health system, including the different committees representing communities. The introduction of automated payment systems and issuance of receipts, especially at PHCs in Enugu, will be a useful practical step to help reduce informal payments.

## Conclusion

This study has described the nature of informal payments, their drivers and the implications for equity in MCH services delivered within primary care. It demonstrates how they have become routine, with severe consequences for those on low incomes, who are being excluded from essential services if they cannot pay. Even what many would consider as very small payments can impact severely on the poorest and most vulnerable women, a group whose health needs are the greatest. We have shown that informal payments are the result of multiple institutional drivers at the facility and health system levels, with health workers demanding payments both to facilitate the operation of the health facilities, as well as to enrich themselves. Understanding the pattern and institutional drivers of informal payments is crucial in developing targeted policies to improve financial risk protection for the poorest, and this in turn is an essential step in improving equity of access to MCH services.

## Data Availability

The data are not available for sharing because of the challenge of fully anonymizing the transcripts and field notes, and taking the sensitive nature of informal payment and ethical restrictions into consideration.

## Reflexivity statement

The authors include four female and two male professionals from Nigeria and the United Kingdom with different levels of seniority and expertise. Four of the authors are professors specialized in health systems policy, accountability and governance, public health, anti-corruption strategies and health economics in West Africa and Europe. Two of the authors are social scientists—an associate professor and an associate lecturer, with expertise in medical anthropology, sociology, social and organizational polices for health determinants, psychology, gender, and health system and policy research. All authors have extensive experience in conducting qualitative (IDI, FGD and ethnography) and quantitative research. The social scientists have done fieldwork in Malawi and Nigeria, especially in southeastern and northern states—Enugu, Anambra, Ebonyi, Kano, Rivers and the Federal Capital Territory Abuja. The study was conducted in a collaborative manner, involving weekly conversations between all team members, enabling them to gradually advance the analysis and achieve an interdisciplinary perspective.
