# Correction to: The nature, drivers and equity consequences of informal payments for maternal and child health care in primary health centres in Enugu, Nigeria

**DOI:** 10.1093/heapol/czae058

**Published:** 2024-07-09

This is a correction to: Pamela Adaobi Ogbozor, Eleanor Hutchinson, Catherine Goodman, Martin McKee, Obinna Onwujekwe, Dina Balabanova, The nature, drivers and equity consequences of informal payments for maternal and child health care in primary health centres in Enugu, Nigeria, *Health Policy and Planning*, Volume 38, Issue Supplement_2, November 2023, Pages ii62–ii71, https://doi.org/10.1093/heapol/czad048

In the originally published version of the manuscript there was an error in the section **Acknowledgements/Funding**. First sentence should read: ‘This work was supported by a research grant from the Health Systems Research Initiative with funding from the UK Foreign, Commonwealth & Development Office (FCDO), the Medical Research Council (MRC) and Wellcome, with support from the UK Economic and Social Research Council (grant number MR/T023589/1).’ instead of ‘This work was supported by a research grant from the Health Systems Research Initiative with funding from the UK Department for International Development, the UK Medical Research Council and the Wellcome Trust with support from the UK Economic and Social Research Council (grant no. MR/T023589/1)’.

The emendation has been made to the article.

